# Changes in the survival of patients with breast cancer: Poland, 2000–2019

**DOI:** 10.1007/s10549-022-06828-5

**Published:** 2022-12-12

**Authors:** Florentino Luciano Caetano dos Santos, Irmina Maria Michalek, Urszula Wojciechowska, Joanna Didkowska

**Affiliations:** Polish National Cancer Registry, Maria Sklodowska-Curie National Research Institute of Oncology, ul. Wawelska 15B, 02-093 Warsaw, Poland

**Keywords:** Breast cancer, Cancer, Survival rate, Epidemiology, Adult, Cancer registries, Population surveillance

## Abstract

**Purpose:**

The main aim of this study was to estimate breast cancer survival in Poland over the period from 2000 to 2019 in both sexes.

**Methods:**

Data were obtained from the Polish National Cancer Registry. The presented metrics included age-standardized 5- and 10-year net survival (NS), median survival times, years of life lost (YLLs), and standardized mortality ratios (SMRs).

**Results:**

Between 2000 and 2019, 315,278 patients (2353 men and 312,925 women; male-to-female ratio 1/100) were diagnosed with breast cancer in Poland. In this period, 721,987 YLLs were linked to breast cancer. Women presented a higher 5- and 10-year age-standardized NS than men (5-year NS: 77.33% for women and 65.47% for men, *P* < 0.001, common language effect size (CL) 1.00; 10-year NS: 68.75% for women and 49.50% for men, *P* < 0.001, CL 1.00). Between the earliest and latest studied period, namely 2000–2004 and 2015–2019, there was a statistically significant increase only in female survival (+ 7.32 pp, *P* < 0.001, CL 1.00). SMRs were significantly higher for women than for men (3.35 vs. 2.89, respectively).

**Conclusion:**

Over the last two decades, breast cancer survival in Poland has improved significantly. Nonetheless, special attention should be given to the disparities between sexes and the gap in overall improvement of survival rates compared with other European countries.

## Background

Breast cancer is the most common cancer globally and the fifth most frequent cause of cancer-related deaths, taking the first position when analyzing the cause of death only in women [1, 2]. Although the incidence of breast cancer has increased over the last few decades, global mortality due to breast cancer has been decreasing. This decrease is due to improvements in treatment, management, and earlier diagnosis through effective population-based screening programs [3–7].

Effective national cancer control strategies aim to address the rising cancer burden and accomplish the 2030 United Nations (UN) Sustainable Development Goals to reduce early mortality due to noncommunicable diseases. As a result, in 2020, the National Strategy for Oncology 2020–2030 plan was approved by the Polish parliament [8]. The plan directs national efforts to fulfill UN goals by improving evidence-based prevention, population screening programs, effective treatments, and palliative care for patients with cancer. The program intends to increase cancer survival and promote cancer epidemiological monitoring through registries that store, present, and analyze data to prioritize and track cancer control efforts.

This study aimed to evaluate the current health status of Polish patients with breast cancer, both men and women, and the time trends of their survival during the last two decades.

## Material and methods

### Source of data

Breast cancer data were obtained from the Polish National Cancer Registry (PLCR), a national institution responsible for epidemiological cancer research in Poland (population of 38.2 million in June 2021 [9]). The PLCR covers practically all incident cancer cases in the Polish population and is the leading contributor of data to cumulative European estimations, representing the largest cancer registry covering the entire national population in the European Union. The operating principles of PLCR have been thoroughly discussed elsewhere [10]. The reports on cancer cases are submitted to PLCR by physicians and coded according to the International Statistical Classification of Diseases and Related Health Problems, Tenth Edition (ICD-10). At PLCR, each reported cancer case is verified by qualified coders based on histopathological/ cytological/ cytometry exam results and coded by applying the International Classification of Diseases for Oncology, Third Edition (ICD-O-3). In 2019, approximately 93% of the cases registered by PLCR were verified morphologically. Each registered case is passed through validation tools to confirm the validity of the cause of death. The PLCR registration system is based on a unique Polish personal identification number (PESEL), averting data duplication.

### Identification of cancer cases

The PLCR database was queried to create a study population that included all Polish individuals diagnosed with breast cancer (ICD-10 code C50) between the 1st of January 2000 and the 31st of December 2019. Vital status was verified in the PESEL database until the 31st of December 2019, during the yearly controls of the registered patients’ vital status. Cases identified solely by death certificate, > 99 and < 15 years of age at diagnosis, without PESEL, or with a follow-up < 30 days after diagnosis were excluded from the analysis. Around 9000 cases were excluded from the analysis due to at least one exclusion criterion.

PLCR is a population-based cancer registry that employs unique identifiers for registration and yearly queries of the PESEL database for follow-up. Therefore, there is no risk of loss to follow-up in the strict sense. Permanently emigrated individuals with no legal ties to the country may account for a small proportion of under-reported deaths. Nonetheless, there is no evidence that such individuals differ significantly in terms of survival and would introduce emigrant bias.

### Statistical analysis

Descriptive statistics were presented for numerical variables as median and interquartile range (IQR) and as numbers and percentages for categorical variables. Depending on the number of samples, the Jarque–Bera test or Shapiro–Wilk test was used to assess the normality of the continuous variables.

To assess the influence of breast cancer on mortality risk, we used standardized mortality ratios (SMRs). SMRs were computed using indirect standardization by age, sex, and year of diagnosis.

Patients were followed up from cancer diagnosis until death, the 31st of December 2019, or the end of 10 years of observation, whichever occurred first. Five and ten-year age-standardized net survival (NS) was estimated by deploying the life table method [11] and the Pohar-Perme estimator [12]. Calculations were conducted for the entire study period overall, four periods of diagnosis (2000–2004, 2005–2009, 2010–2014, and 2015–2019), and by age at diagnosis groups. The cohort approach was deployed for most of the periods, except for (2015–2019) when complete follow-up data were unavailable for all individuals, and the period analysis method [13] was used. Age-standardized NS estimates were calculated using the International Cancer Survival Standard (ICSS) weights. Age at diagnosis was categorized into five groups, namely 15–44, 45–54, 55–64, 65–74, and 75 + years, following the ICSS guidelines. The corresponding 95% confidence intervals (95% CI) were estimated using log transformation. The between-group survival difference by time interval was assessed using a two-tailed test and between median measures (such as median age at diagnosis with the Kruskal–Wallis rank-sum test). To discard the potential significance bias due to the large sample size, we deployed size Hedges’ g, which was followed by transformation into common language effect size (CL). CL expresses the Hedges’ g effect size, also known as the standardized mean difference, reflecting the likelihood that a random observation from one population would be larger than another random observation from another population [14]. Due to the presence of medians (representing non-normal distributions), for the approximate mean and standard deviation (SD) calculation, we applied the methods proposed by Wan et al. [15]. The associations were only considered significant at the overall alpha level set at < 0.05 and with a large CL (> 0.80) [14].

Kaplan–Meier survival curves were generated to estimate survival rates at a specific time after diagnosis, and the corresponding 95% CIs were computed using log transformation. Multivariate Cox proportional hazards regression models were fit to generate mortality hazard ratios (HR) and the corresponding 95% CIs, to describe the association between exposures (age at diagnosis, sex, and year of diagnosis) and time-to-event (death). A two-sided log-rank test was used to assess the significance of the exposure. HRs that include 1.0 in their 95% CI range were deemed irrelevant.

The cause-specific years of life lost (YLLs) were calculated using age-specific life expectancy tables for the Polish population. The reference age was the average life expectancy at birth between 1950 and 2019, namely 67.4 years for men and 74.9 years for women, according to Statistics Poland [16]. Individuals diagnosed after this age were excluded from the YLLs analysis. YLLs’ 95% CIs were produced using bootstrapping with 500 resamples from the cohort population. YLLs that include 0.00 in the 95% CI range were deemed not meaningful.

All above statistical analyses were performed using RStudio version 1.4.1103 (R Foundation for Statistical Computing, Vienna, Austria). Lifetables specific to the Polish population were obtained from Statistics Poland [16].

To determine survival trends and generate percentage change (PC) with 95% CI, joinpoint regression was applied. The best-fitting model was selected with permutation tests, with an overall significance level of 0.05, and the number of randomly permutated datasets for permutation was set at 4499. Rates were considered to decrease if PC < 0 and 95% CI did not contain zero and increased if PC > 0 and 95% CI did not contain zero; otherwise, rates were considered stable. Joinpoint analysis was performed using Joinpoint Regression Program (version 4.7.0.0, National Cancer Institute, Bethesda, MD, USA).

### Compliance with ethical standards

The PLCR obeys strict regulations to secure the complete confidentiality and protection of individuals, and individual-level data can be used for statistics in aggregate form for scientific purposes, according to Polish law. This study was conducted according to the Strengthening the Reporting of Observational Studies in Epidemiology (STROBE) guidelines [17].

## Results

### Patients’ characteristics

For the period 2000–2019, we included 315,278 cases of breast cancer (2353 men, 312,925 women; male-to-female ratio 1/100). Table 1 presents the number of incident cancer cases according to the diagnosis period. The median age at diagnosis was 60 years (IQR = 18 years), with no statistically significant and large effect size differences between men and women (men: 66 years, IQR = 16 years; women: 60 years, IQR = 17 years; *P* < 0.001; CL = 0.63). The sex- and age-standardized SMR for breast cancer in men was 2.89 (95% CI = 2.72 to 3.06) and for women 3.35 (3.33 to 3.37), with a statistically significant and high effect size (*P* < 0.001; CL = 1.00).

**Table 1:** Number of incident breast cancer cases by period of diagnosis, 5- and 10-year age-standardized net survival, and age-standardized cause-specific years of life lost (YLLs) with respective 95% confidence intervals (95% CI), by sex—Poland, 2000–2019

Sex	Number of cases					Average YLL (95% CI) [years]		Cumulative YLL [years]	Age-standardized net survival^a^ (95% CI)				
	Total	2000–2004	2005–2009	2010–2014	2015–2019				5-year	10-year	Difference	*P*-value	*CL*
Men	2353	487	533	628	705	3.93 (3.53–4.24)	*τ* = *67.4*	9247	65.47% (62.35–68.40%)	49.50% (44.65–54.16%)	−15.97 pp (−16.20 to −15.74 pp)	*P* < 0.001	1.00
Women	312,925	62,174	73,817	84,607	92,327	5.06 (5.02–5.09)	*τ* = *74.9*	1,583,401	77.33% (77.06–77.59%)	68.75% (68.29–69.22%)	−8.58 pp (−8.58 to −8.58 pp)	*P* < 0.001	1.00
						*P* < 0.001			*P* < 0.001	*P* < 0.001	*P* < 0.001		
						CL 1.00			CL 1.00	CL 1.00	CL 1.00		

^a^2000–2014 cohort approach and 2015–2019 period analysis

### Survival analysis for the time of the study

In the last period of analysis (2015–2019), the age-standardized 5-year NS for male breast cancer was 68.67% (95% CI = 63.43–73.31%) and for female breast cancer was 80.40% (79.97–80.81%). The differences between the sexes were statistically significant (*P* < 0.001; CL = 1.00; Table 2).

**Table 2:** Age-standardized 5-year net survival and 95% confidence intervals (95% CI) for breast cancer by period of diagnosis and sex—Poland, 2000–2019

Sex	Age-standardized 5-year net survival^a^ (95% CI)						
	2000–2004	2005–2009	2010–2014	2015–2019	Difference 2000–2004 vs. 2015–2019	P-value	CL
Men	63.12%	66.33%	65.70%	68.67%	+ 5.55 pp	< 0.001	0.76
	(56.42–69.08%)	(60.05–71.85%)	(60.26–70.58%)	(63.43–73.31%)	(+ 4.88 to + 6.22 pp)		
Women	73.08%	76.11%	78.81%	80.40%	+ 7.32 pp	< 0.001	1.00
	(72.47–73.69%)	(75.59–76.62%)	(78.36–79.25%)	(79.97–80.81%)	(+ 7.31 pp to + 7.33 pp)		
*P*-value	< 0.001	< 0.001	< 0.001	< 0.001			
CL	1.00	1.00	1.00	1.00			

^a^2000–2014 cohort approach and 2015–2019 period analysis

For the entire period of analysis (2000–2019), the overall age-standardized 5-year NS for men was 65.47% (95% CI = 62.35–68.40%) and for women 77.33% (77.06–77.59%), and the overall age-standardized 10-year NS for men was 49.50% (44.65–54.16%) and women 68.75% (68.29–69.22%). The differences were statistically significant for both the 5- and 10-year NS, with a high effect size (*P* < 0.001; CL = 1.00). The difference between the 5- and 10-year male NS was −15.97 percentage points (pp; 95% CI −16.20 pp to −15.74 pp; *P* < 0.001; CL = 1.00), and female NS −8.58 pp (−8.58 pp to −8.58 pp; *P* < 0.001; CL = 1.00). The median survival time for male breast cancer was 5.64 years (95% CI = 5.25–6.06 years). As women did not achieve 50% of the initial cohort size, their median survival time could not be calculated (Table 1). There was a statistically significant decrease in the HR associated with female sex (HR 0.57; 95% CI = 0.54–0.61; *P* < 0.001).

Between 2000 and 2019, there were 1,592,648 YLLs caused by breast cancer. Women contributed 1,583,401 YLLs and men 9247 YLLs (less than 1%). The average YLL due to breast cancer was 3.93 years (95% CI = 3.53–4.24 years) for men and 5.06 years (5.02–5.09) for women, with significant difference between the sexes (*P* < 0.001; CL = 1.00; Table 1).

### Survival analysis by year of diagnosis

**Fig. 1:**
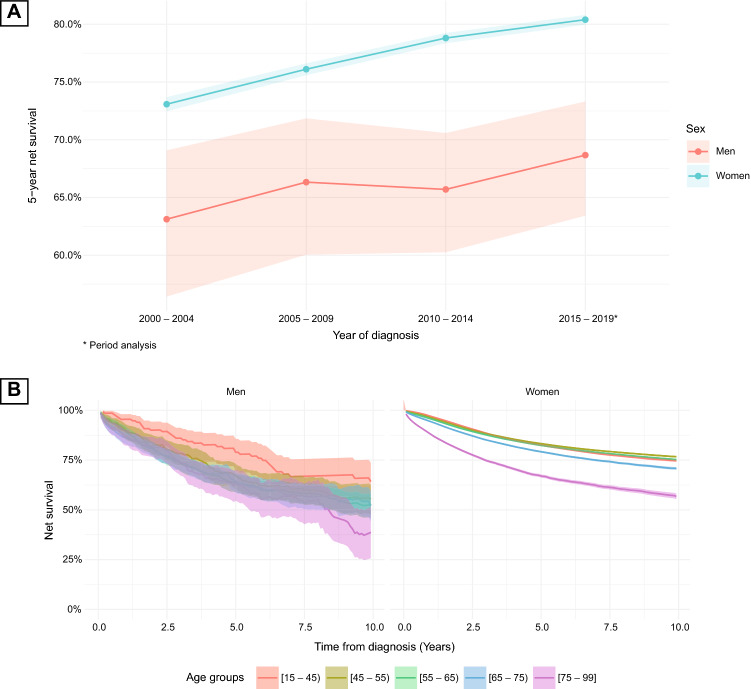
Panel **A** Age-standardized 5-year net survival for breast cancer by period of diagnosis and sex—Poland, 2000–2019. Panel **B** Net survival for breast cancer, by age group and sex—Poland, 2000–2019

Between the first and last analysis periods, the age-standardized 5-year NS for breast cancer increased in both sexes. The increase among women was statistically significant at 7.32 pp (from 73.08 to 80.40%; *P* < 0.001; CL = 1.00), whereas that among men was insignificant at 5.55 pp (from 63.12 to 68.67%; *P* < 0.001; CL = 0.76). There were significant sex-dependent differences in survival, for all periods of diagnosis, with a large effect size (*P* < 0.001; CL = 1.00; Table 2 and Fig. 1A). To further assess survival time trends, we fitted joinpoint regressions to the 5-year NS for both sexes according to the period of diagnosis. In women the survival time trend was significantly ascending with PC of 0.6 (95% CI 0.4–0.8) and with no joinpoints. In men survival time trend was stable with PC of 0.5 (95% CI −0.2 to 1.2). A significant increase in HR was associated with the year of diagnosis for the entire study cohort (HR 1.03; 95% CI = 1.02–1.04; *P* < 0.001).

### Survival analysis by age at diagnosis

Overall, both the 5- and 10-year NS decreased with age at diagnosis independently of sex. For men, between the age groups [15–45 years] and [45–55], there was a decrease in NS of around 13 pp. It was followed by a plateau between [45–55], [55–65], and [65–75] age groups. Finally, there was a sharp decrease in the [75–99] age group, especially in the 10-year NS (14 pp). For women, between the age groups [15–45] and [45–55 years], there was a significant increase of both 5- and 10-year NS survival, with a high effect size (5-year NS: from 82.70 to 83.46%; *P* < 0.001; CL = 0.94; 10-year NS: from 74.65 to 76.68%; *P* < 0.001; CL = 1.00). This was followed by a decline in survival in the following age groups (Table 3 and Fig. 1B). There was no statistically significant association between age at diagnosis and NS for the entire cohort (HR 0.99, 95% CI = 0.98 to 1.00, *P* = 0.088).

**Table 3:** Age-standardized 5- and 10-year net survival and 95% confidence intervals (95% CI) for breast cancer by age group and sex—Poland, 2000–2019

Age group	Number of cases		Age-standardized net survival (95% CI)							
			5-year				10-year			
	Men	Women	Men	Women	*P*-value	CL	Men	Women	*P*-value	CL
[15–45)	127	36,935	80.95% (72.05–87.26%)	82.70% (82.26–83.12%)	< 0.001	0.98	64.21% (52.62–73.66%)	74.65% (74.09–75.19%)	< 0.001	1.00
[45–55)	285	73,820	68.03% (61.34–73.81%)	83.46% (83.15–83.76%)	< 0.001	1.00	55.38% (47.47–62.59%)	76.68% (76.29–77.06%)	< 0.001	1.00
[55–65)	654	91,051	63.86% (59.08–68.24%)	82.54% (82.23–82.83%)	< 0.001	1.00	52.13% (45.92–57.97%)	75.27% (74.86–75.69%)	< 0.001	1.00
[65–75)	754	66,792	65.06% (59.84–69.77%)	79.37% (78.94–79.78%)	< 0.001	1.00	52.24% (44.46–59.45%)	70.82% (70.17–71.46%)	< 0.001	1.00
[75–99]	533	44,327	62.37% (54.02–69.63%)	67.33% (66.56–68.09%)	< 0.001	1.00	38.69% (25.67–51.53%)	56.81% (55.38–58.22%)	< 0.001	1.00

## Discussion

### Net survival in the context

We estimated age-standardized relative survival and other health metrics for breast cancer, which accounted for more than 22.9% of female incident cancer cases and 15.1% of female deaths due to cancer in Poland in 2019 [10]. Between 2000 and 2019, breast cancer survival in Polish patients improved, independent of sex (5-year NS: 5.55 pp for men and 7.32 pp for women).

During the study period, Poland steadily approached the breast cancer survival rates of Norway and Finland [18], which are regarded as leaders of high-quality healthcare provision in Europe. Although the gap in survival is slowly closing, Poland is still lagging behind Nordic countries by approximately 10 pp (10.7 pp with Finland and 11.6 pp with Norway [19, 20]). When compared with neighboring countries and the former communist bloc, Poland also lags, but on a much smaller scale (difference in women: 6.3 pp from Czechia, 5.6 pp from Germany, and 1.6 pp from Estonia). Survival in male breast cancer in Poland is lower than that in Germany (2.7 pp), but higher than that in Estonia (7.7 pp; 21–24). No comparisons can be made regarding Nordics, since the only available report on male breast cancer survival was a Finnish single-center study (5-year observed survival was of 75% [21]), which was not generalizable.

Recently published analyses on the links between breast cancer NS and age at diagnosis are scarce, pertain to women only, and encompass Germany, Norway, and Finland. Compared to these countries, Poland still presents a lower NS in all age groups studied. The difference increased with age at diagnosis and was especially noticeable in the 75 + years age group (average difference of: 15–45 group = 9.7 pp, 45–55 group = 10.0 pp, 55–65 group = 10.8, 65–75 group = 12.0, and 75 + group = 14 pp, [19, 20]).

Differences in breast cancer survival between countries may be due to different profiles of risk factors, including age at diagnosis, menarche history, parity, hormone use, alcohol and tobacco use, and physical activity. It is noteworthy that tobacco usage in Poland has been declining in recent decades, which might positively influence the incidence of cancers and overall population health metrics [22–24].

Access to breast cancer screening programs aimed at early detection and treatment also plays a major role in the survival landscaping. A variable that is somewhat connected to screening programs and might partially explain the country differences in incidence and survival is the prevalence of genetic mutations in populations; for example, mutations in BRCA 1 and BRCA2 tumor suppressor genes, which are significantly associated with the development of breast cancer by the age of 70 years [6]. Due to the early onset and few early symptoms of this type of breast cancer, it is crucial to provide special pathways in screening programs for potential mutation carriers, following the recommendations of the European Commission Initiative on Breast Cancer Guidelines Development Group [4].

Finally, differences in survival metrics may vary by country because of differences in access to advanced therapeutic options. The increase in survival reported in this study may be partially attributed to the advancements in breast cancer treatment. Over the last 15 years, breast cancer treatment has shifted to a more personalized treatment, based on targeted therapies (hormone receptor-based, HER2 status-based, BRCA mutation-targeted, and CDK4/6 inhibitors) and immunotherapy (PD-1 inhibitor, antibody–drug conjugate T-DM1) [25–27], bolstered by novel diagnostic techniques, such as gene expression profiling [28].

### Disparities of net survival between sexes

In Poland women have higher breast cancer survival rates than men, with difference in 10-year NS at 19.25 pp (95% CI = 19.06 pp to 19.44 pp; *P* < 0.001; CL = 1.00). The observed sex-associated survival disparity concords with previously published studies and cancer registry reports from other countries [29–32].

Age at diagnosis cannot explain these disparities since there was no significant association between age at diagnosis and NS. A potential explanation for the observed differences could be the cancer staging at diagnosis. It is well established that male breast cancer diagnosis is challenging [33], what is a resultant of lack of knowledge, public education, and embarrassment delaying the diagnoses. Nevertheless, previous studies have refuted this hypothesis for most solid tumors, for which even after adjusting for sex, age, and stage, female survival rates were still higher than those in male [34, 35]. Other plausible explanations include sex-associated variations in genetic factors, different therapy responses and compliance, concurrent neoplasms, hormone use, alcohol and tobacco use, physical activity, family history of cancer [36–39], and other etiological factors [1, 5, 6, 30, 40–42]. Finally, the last criterion that might influence both the incidence and survival statistics of male breast cancer is sex reassignment, either male-to-female or female-to-male. Previous studies have indicated that hormonal stimulation linked to sex reassignment might induce breast cancer development [43, 44].

Breast cancer in men is still understudied due to several factors, such as lack of concern due to low prevalence (< 1% of all breast cancers globally) or difficulties in establishing a cohort that allows the calculation of viable and representative metrics. The latter can only be tackled by performing multinational studies, such as the Global Burden of Disease project, which uses estimates and modeling to assess risks, predictions, and spatial distributions, or by population cancer registries that use real-life data, such as the PLCR. Future research should focus on examining the connection between sex, potential risk factors, and breast cancer survival.

### Other metrics in the context

Comparison of HRs, YLLs, median survival times, and SMRs for the studied cancers with previous literature for neighboring countries was impossible due to several factors, such as the lack of previously published reports or different time intervals, statistical methods, and standards employed, especially for male breast cancer. Also, as Poland has reached the “above five years” median survival times, it was impossible to compare it with the previously mentioned cancer registry reports [20, 29, 45, 46]. Nonetheless, these metrics are valuable for assessing the increased risk of death between sexes (HRs and median survival times) and compared with the general population (YLLs and SMRs). Even though our results showed a significant increase in NS for women (also depicted by the HR of 0.57 when compared with men), the age- and sex-standardized SMR continues to be higher for this sex (3.35 for women vs. 2.89 for men) compared with the general population. This can potentially be associated with a higher life expectancy in cancer-free women than in cancer-free men. The same interpretation can also explain the higher YLLs in women.

### Suggestions for the future research work

Although the survival rates for breast cancer have increased in the last 20 years, if the gaps between countries are to be closed, further work is required.

Future studies should identify proper target populations for intervention and the potential expansion of screening programs. It is also important to determine the influence of demographic, social, economic, and environmental factors on breast cancer survival. When provided with a proper and more in-depth analysis, policymakers and other stakeholders can trigger necessary reforms to positively impact breast cancer survival and survivorship.

Other topics to be covered by future studies should consider the weight and impact of the COVID-19 pandemic on breast cancer survival, an effect that may only be comprehensively assessed in several decades. Also, due to the forced migration of over a one and a half million Ukrainian women into Poland (state for 20th of April 2022), it is expected that the incidence, prevalence, and survival of breast cancer will shift within the next decade. To reduce the influence of this extraordinary strain of forced migration on the Polish healthcare system, special financial aid programs should be implemented at the international level.

Finally, due to the overcoming of the 5-year survival in most countries, it would be plausible to start analyzing the survival of breast cancer not in the canonical 5- and 10-year follow-up time but by extending it to 20 or even further, or to consider the patient cured of breast cancer after a certain period.

### Limitations and strengths of the study

One potential limitation of our study was that the number of male patients was small, even though our cohort was large. Although this relatively small sub-cohort might influence the results, using statistical effect size metrics, such as Hedges’ g and its CL, which analyzes the size of the difference between classes, allows weighting and strengthens the decision on the statistical significance and relevance of the obtained metrics, especially when analyzing sex differences.

The main strengths of this study are the large study population with high national coverage of cancer cases, providing representability and generalizability of the obtained metrics, a long follow-up period (20 years), presentation of metrics for male breast cancer with a relatively high number of cancer cases (rarely reported in epidemiological studies), and a high percentage of morphologically verified cancer cases (during the study period this percentage increased from 65 to 92% and the mortality-to-incidence ratio changed from around 0.7 to 0.5). Improvements in the fraction of morphologically verified cases and the mortality-to-incidence ratio reflect an improvement in cancer registration and data quality throughout the study period, potentially underestimating the survival rates observed. Finally, since governmental institutions subsidize the Polish healthcare system, potential biases due to unaffordability of healthcare services can be excluded, as access to diagnostic and treatment measures is equal regardless of socioeconomic status.

## Conclusions

The survival of patients with breast cancer in Poland has increased considerably over the last two decades. There were evident discrepancies between the sexes, with women presenting a higher NS than men. Although breast cancer survival has increased, special consideration should be given to diminishing the survival gap compared to other European countries. Screening programs should be extended to especially vulnerable groups, and further analyses should be conducted to contribute to our understanding of sex disparities in survival rates.

## Data Availability

The data analyzed in this study were obtained from the Polish National Cancer Registry and are available upon reasonable request and subject to ethical approval in place and material transfer agreements.
